# Differences in maternal and perinatal outcomes between Dutch and non-Western women in a midwife-led care setting: a retrospective cohort study

**DOI:** 10.1186/s12884-024-06982-2

**Published:** 2024-11-29

**Authors:** F. Ali, L. A Horvat – Gitsels, P. C. A. M Bakker, C. J. M. Verhoeven, J. T. Gitsels- van der Wal

**Affiliations:** 1 Department of Obstetrics and Gynaecology, Amsterdam UMC Location Vrije Universiteit Amsterdam, De Boelelaan 1117, Amsterdam, The Netherlands; 2 UCL Great Ormond Street Institute of Child Health, 30 Guilford St, London, WC1N 1EH UK; 3 Moody’s Corporation, The Minster Building, 21 Mincing Lane, London, EC3R 7AG UK; 4 Midwifery Science, Amsterdam UMC Location Vrije Universiteit Amsterdam, De Boelelaan 1117, Amsterdam, The Netherlands; 5 Division of Midwifery, School of Health Sciences, University of Nottingham, Nottingham, UK; 6 Amsterdam Public Health, Quality of Care, Amsterdam, The Netherlands; 7 Midwifery Academy Amsterdam Groningen, Inholland, Vlaardingenlaan 1, 1059 GL Amsterdam, The Netherlands; 8 Department of Primary and Long-term Care, University Medical Center Groningen, PO Box 196, 9700 AD Groningen, The Netherlands; 9 Department of Obstetrics and Gynaecology, Maxima Medical Center, De Run 4600, 5504 DB Veldhoven, The Netherlands; 10 Amsterdam Reproduction and Development research institute, Amsterdam, The Netherlands

**Keywords:** Midwifery, Perinatal outcomes, Ethnicity, Perineum, Birthweight, Cesarean section, Low-risk women

## Abstract

**Background:**

Previous research has shown that genetics and maternal medical, sociodemographic, lifestyle and psychosocial factors affect maternal and perinatal outcomes. Substantial research has been done on ethnic differences and maternal and perinatal outcomes in hospital settings. To our knowledge there are no studies about the associations between ethnicity and maternal and perinatal outcomes in a midwife-led care setting among low-risk women. Therefore, our study aimed to investigate possible ethnic associations between non-Western and Dutch women, and maternal and perinatal outcomes in a midwife-led care setting.

**Methods:**

A retrospective cohort study was performed of low-risk pregnant women (*n* = 977) in midwife-led care. Data was collected from a medium-sized midwifery practice in an urban region near Amsterdam, the Netherlands. Regression analyses were performed to examine the effect of ethnicity on maternal and perinatal outcomes. Outcomes of interest were gestational age, mode of birth, perineal status, postpartum hemorrhage, birthweight, perinatal death and low Apgar score. Associations were corrected for deprived areas, body mass index (BMI), parity and maternal educational level. Potential effect modification for prenatal referral to obstetrician and parity were assessed.

**Results:**

The study included 977 women, of whom 483 were non-Western, and 494 were Dutch. Regarding characteristics, compared to Dutch women, non-Western women were more likely to be multiparous (respectively 58.6% versus 49.2%; *p* = 0.003), live in a deprived area (34.0% versus 8.1%; *p* < 0.001), have limited formal education (medium: 46.0% versus 49.2%; low: 15.6% versus 7.4%; *p* < 0.001), have a higher BMI (overweight: 28.6% versus 22.9%; obese: 14.9% versus 12.0%; *p* = 0.045), make inadequate/intermediate use of prenatal care (7.2% versus 2.4%, *p* < 0.001) and suffer from gestational diabetes (17.2% versus 9.9%, *p* < 0.001). Whereas Dutch women were more likely to suffer from psychosocial problems during and/or before pregnancy (34.8% versus 23.0%, *p* < 0.001) and drink alcohol during pregnancy (5.9% versus 1.9%, *p* = 0.001). Regarding maternal and perinatal outcomes, non-Western women had increased odds of perineal laceration (OR 1.59, 95%CI 1.14–2.21) and decreased odds of high birthweight (0.50, 95%CI 0.29–0.84). The mode of birth differed by ethnicity. The interaction of prenatal referral and ethnicity was significant for the mode of birth. Therefore, for mode of birth the groups were stratified by prenatal referral (yes/no). In the prenatally referred group (*n* = 474), non-Western ethnicity was significantly associated with decreased odds of cesarean Sect. (0.63, 0.40–0.98). No other associations were significantly associated with ethnicity.

**Conclusions:**

Maternal and perinatal outcomes differed between low-risk non-Western and Dutch women in a midwife-led care setting. Among non-Western women, perineal laceration occurred more often, and fewer children with high birthweight were born. In the prenatally referred group, women of non-Western ethnicity had decreased odds of cesarean section. Gestational age and postpartum hemorrhage were not significantly associated with ethnicity.

**Supplementary Information:**

The online version contains supplementary material available at 10.1186/s12884-024-06982-2.

## Background

Multiple studies conducted in Western countries show that poorer perinatal health disproportionally burdens non-Western women and their offspring [1–3]. A common finding in these studies is that non-Western women, are more likely to experience adverse maternal and perinatal outcomes, e.g. preterm birth, compared to Western women [4]. However, not only ethnicity plays a role in this disparity, but possible related factors to ethnicity such as maternal medical, sociodemographic, lifestyle and psychosocial factors as well [4–7].

Various studies have shown that non-Western women are less likely to make adequate use of care during pregnancy (prenatal/antenatal care), compared to Western women. According to a systematic review conducted by Boerleider et al. 2013, language barriers and limited knowledge of the Western healthcare system were most commonly noted factors that inhibit non-Western women to make adequate use of prenatal care [8] Boerleider et al. 2014 also conducted a prospective study into explanatory factors for inadequate use of prenatal care by non-Western women in a midwife-led setting. Non-Western women were again more likely to make less use of prenatal care due to socioeconomic factors [9]. And as Boerleider et al. 2014 further states in the paper, prior research has shown that inadequate use of prenatal care can lead to adverse pregnancy outcomes [9]. A recent study conducted by [10] on obstetric care for refugees in the Netherlands showed that next to language barriers and limited knowledge of Western health care, there is also (often unconscious) racism from healthcare providers [10]. This not only results in poorer perinatal care for the pregnant women but may also delay or entirely discourage the seeking of prenatal care [10]. And this is concerning, as prenatal care is primarily conducted to identify and/or prevent adverse outcomes in a timely manner [11].

The association between ethnicity and maternal and perinatal outcomes in Western countries has been studied before. Zwart et al. assessed the ethnic differences in severe acute maternal morbidity (SAMM) in the Netherlands; they found that non-Western women had a higher chance of experiencing SAMM, partially due to differences in maternal factors, such as socioeconomic and lifestyle-related factors [3]. Similar research in a Dutch population on preterm birth found that non-Western women are more likely to give birth preterm because of the accumulation of various maternal factors such as smoking and depression [2]. Ethnic disparities are also observed in the mode of birth. Non-Western women were more likely to have a cesarean section and consequently suffer from cesarean-related morbidities, such as postpartum hemorrhage and uterine rupture [1, 12].

The aforementioned research has been done on ethnic differences in outcomes in obstetrician-led care and thus among high-risk women or no distinction was made between obstetrician-led care or midwife-led care. Previous research has shown that midwife-led care for low or medium-risk pregnant women is associated with better maternal and perinatal outcomes compared to obstetrician-led care [13–15]. For example, in midwife-led care, pregnant women were less likely to have an intervention during birth than in obstetrician-led care [13]. Furthermore, adverse outcomes such as postpartum hemorrhage were not found to be higher in midwife-led care compared to obstetrician-led care [13]. To our knowledge there are no studies about the associations between ethnicity and maternal and perinatal outcomes in a midwife-led care setting among low-risk women. Therefore, this paper aimed to investigate whether ethnicity is associated with maternal and perinatal outcomes among low-risk women in a midwife-led care setting.

## Methods

### Study design

This retrospective cohort study used data from a medium-sized midwifery practice that takes care of approximately 750 low-risk pregnant women annually [16]. The midwifery practice is in a city close to Amsterdam, the Netherlands. Data collection was from January 2015 to January 2017. The study was approved by the Medical Ethics Committee of the Amsterdam University Medical Center (ref. 2018.019).

### Participants

Women were eligible for inclusion if they were registered for prenatal care at the midwifery practice and had a singleton pregnancy [16]. Exclusion criteria were miscarriage/ectopic pregnancy of the current pregnancy, prenatal referral to an obstetrician after just one consultation, moved outside region, and unknown ethnicity. All ages were included, including those younger than 18 years (*n* = 3). Informed consent was given verbally and noted in the medical file with the pregnant women present.

### Research setting

In the Netherlands, the type of care a pregnant woman receives is determined through risk selection. The primary care midwife or general practitioner is responsible for the risk selection. They assess the pregnant woman's general, gynecological, and obstetric history to determine where the pregnant women can best receive her care. Independent primary-care midwives care for women with a low risk of pathology. Women with a low-risk pregnancy can opt to give birth at home or in a hospital or birth center, attended by their midwife. In case of an increased risk for adverse maternal or perinatal outcomes, the midwife refers the pregnant woman to specialized care provided in a hospital setting by hospital midwives and obstetricians. Pregnant women can be referred to specialized care during the pregnancy (prenatal), during childbirth (antepartum) or after birth (postnatal) [17]. If the complication is no longer present, the obstetrician can refer the pregnant women back to the midwife.

### Data collection and maternal and perinatal outcomes

Data were obtained from a digital maternity database [16]. Data contained information on the use of prenatal care, sociodemographic and medical characteristics, and maternal and perinatal outcomes of women and their newborns. Maternal outcomes of interest were gestational age at birth (weeks' gestation), mode of birth (spontaneous, vaginal assisted birth, or cesarean section), postpartum hemorrhage (> 1000 ml) and perineal status (intact, lacerated, episiotomy). Perinatal outcomes of interest were birthweight (small for gestational age [SGA]: < 10th percentile for gestational age; average for gestational age [AGA]: 10th-90th percentile; or large for gestational age [LGA]: > 90th percentile) [18], perinatal death and Apgar score at five minutes (< 7/ ≥ 7).

### Determinants

The primary determinant was ethnicity (Dutch/Western non-Dutch/non-Western). For determining ethnicity, we used the definitions provided by Statistics Netherlands. This organization is legally mandated to conduct and publish statistics on various societal topics. In 2021, they introduced a new classification system for ethnicity, which was applied in this study. According to CBS definition, in this study, ethnicity was based on the country of birth. First, if born in the Netherlands, the woman was considered to be of Dutch ethnicity. However, if at least one of her parents were born abroad, the woman was considered to have the same ethnic origin as the non-Dutch parent. Second, the woman was of non-Western ethnicity if she or one of her parents were born in Africa, Latin America, Asia (excluding Japan and Indonesia), or Turkey [19, 20]. Last, if the woman is not Dutch but has a Western ethnicity (Europe, North America, Oceania, Indonesia or Japan), the woman is considered Western non-Dutch [21]. Western non-Dutch women were excluded from the study due to the small size of the group (8%) and the significant cultural and socioeconomic differences for it to be combined with either the Dutch or the non-Western group.

The following covariates were considered, hereafter referred to as maternal characteristics: maternal age (in years), maternal educational level (low: primary school and uncompleted vocational training; intermediate: secondary school and completed vocational training; high: higher professional education or university), deprived area based on postal code (yes/no), parity (nulliparous/multiparous), body mass index (BMI) (underweight {< 18.5}, normal weight {18.5–24.9}, overweight {25.0–29.9}, or obese {≥ 30.0} [22], smoking during pregnancy (yes/no), gestational diabetes (yes/no), pregnancy-induced hypertension (yes/no), uptake of prenatal care (adequate, adequate + , or inadequate/intermediate), prenatal referral to the obstetrician (yes/no) and psychosocial problems (yes in the past/yes in the present/no). The adequacy of prenatal care utilisation was determined using the validated Kotelchuck Index Revised (KI-R) [23, 24]. This index follows the guidelines of the Royal Dutch Organization of Midwives; it is calculated by the start of prenatal care (preferably for 12 weeks), number of face-to-face prenatal visits with a midwife and gestational age [16, 23, 24]. Psychosocial problems were defined as "the broad spectrum of all complaints which are not strictly medical or somatic and affect the patient's functioning in daily life; for example, stress, sleep disorder, relationship problems, financial problems, housing problems and adjustment problems" [16, 25] and treated by a psychologist or psychiatrist. If treatment happened more than five years ago, psychosocial problems were classified as not present.

### Statistical analyses

Analyses were performed in SPSS version 27 [26] Significance level was set at *α* = 0.05. Missing values in the determinants was dealt with by listwise deletion [27]. Missing values in the maternal and perinatal outcomes were dealt with by pairwise deletion to maximise the sample size for each outcome of interest. Patterns of missing data found by logistic regressions may bias the generalizability of the results towards the group overrepresented in the complete sample.

Chi-squared and t-tests assessed differences in maternal characteristics between the two groups, as well as maternal and perinatal outcomes by ethnicity. Regression models of maternal and perinatal outcomes were fitted to assess associations with ethnicity, logistic models for gestational age (< 37 weeks) and postpartum hemorrhage (yes), and multinominal models for mode of birth, birthweight, and perineal status. No regression model was fitted for perinatal death and low Apgar score due to its rarity (< 5% prevalent and n < 30). The regression models were adjusted for significant confounders of maternal characteristics found through forward selection using the change-in-estimate (CIE) criterion [28]. Potential effect modification by prenatal referral to an obstetrician and parity [14, 29] were assessed by adding their interaction terms with ethnicity to the regression model. If an interaction term was significant, the model was stratified by the effect modifier. All models' assumptions were satisfied.

## Results

### Study population

**Fig. 1:**
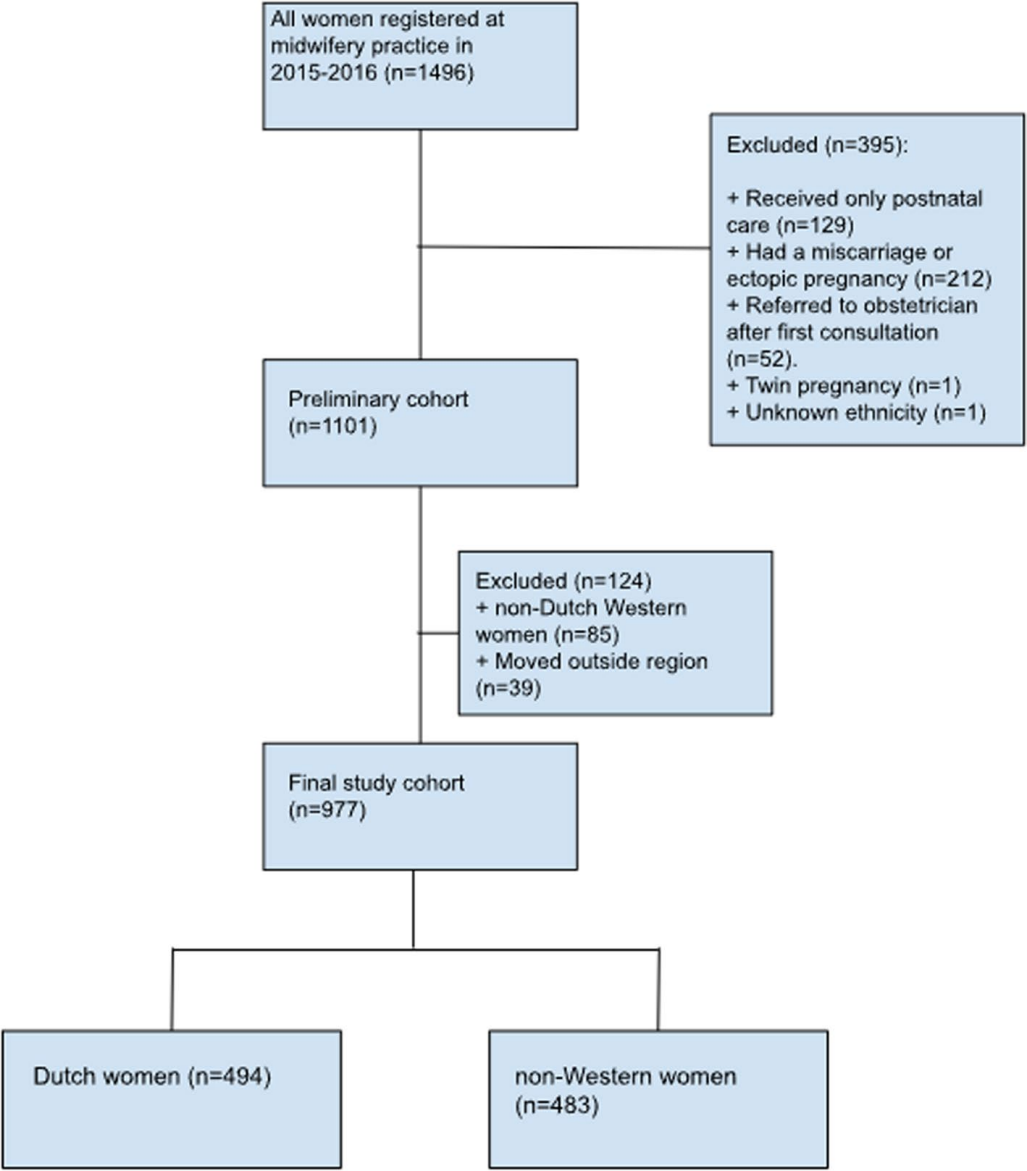
Flow chart of the eligible study population

This final study cohort included 977 women, of whom 51% (*n* = 494) were Dutch, and 49% (*n* = 483) were non-Western (Fig. 1). These groups differed in maternal characteristics (Table 1). Dutch women were more likely to be nulliparous (50.8% versus 41.4% of non-Western women, *p* = 0.003), suffer from psychosocial problems during and/or before pregnancy (34.8% versus 23.0%, *p* < 0.001) and drink alcohol during pregnancy (5.9% versus 1.9%, *p* = 0.001). In contrast, non-Western women made inadequate/intermediate use of prenatal care more often (7.2% versus 2.4%, *p* < 0.001), had limited formal education (medium: 46.0% versus 49.2%; low: 15.6% versus 7.4%; *p* < 0.001), had higher BMI (overweight: 28.6% versus 22.9% or obese: 14.9% versus 12.0%, respectively; *p* = 0.045), lived in deprived area more often (34.0% versus 8.1%, *p* < 0.001), and suffered from gestational diabetes more often (17.2% versus 9.9%, *p* < 0.001).

**Table 1:** Maternal characteristics of Dutch and non-Western women (*N* = 977)

	**Study population *N* = 977 (%)**^a^	**Dutch women *n* = 494 (50.6%)**^a^	**Non-Western women *n* = 483 (49.4%)**^a^	***p*-value**
**Age in years [IQR]**^b^	29 [26–33]	30 [36–33]	29 [26–32]	0.05
**Prenatal care utilization**				
Intermediate/inadequate	47 (4.8)	12 (2.4)	35 (7.2)	< 0.001
Adequate	705 (72.2)	354 (71.7)	351 (72.7)	
Adequate +	225 (23.0)	128 (25.9)	97 (20.1)	
**Prenatal referral to the obstetrician**	474 (48.5)	227 (46.0)	247 (51.1)	0.11
**Level of education**				< 0.001
Low	109 (11.5)	36 (7.4)	73 (15.6)	
Medium	453 (47.6)	238 (49.2)	215 (46.1)	
High	389 (40.9)	210 (43.4)	179 (38.3)	
**Deprived area**	204 (20.9)	40 (8.1)	164 (34.0)	< 0.001
**Parity**				0.003
Nulliparous	451 (46.2)	251 (50.8)	200 (41.4)	
Multiparous	526 (53.8)	243 (49.2)	283 (58.6)	
**Psychosocial problems**	282 (28.9)	171 (34.8)	111 (23.0)	< 0.001
**Smoking**	212 (21.8)	116 (23.5)	96 (20.0)	0.18
**Alcohol use**	38 (3.9)	29 (5.9)	9 (1.9)	0.001
**BMI**				0.045
Underweight	41 (4.2)	24 (4.9)	17 (3.5)	
Normal weight	552 (56.6)	297 (60.2)	255 (52.9)	
Overweight	251 (25.8)	113 (22.9)	138 (28.6)	
Obese	131(13.4)	59 (12.0)	72 (15)	
**Pregnancy-induced hypertension**	68 (7.0)	40 (8.1)	28 (5.8)	0.16
**Gestational diabetes**	132 (13.5)	49 (9.9)	83 (17.2)	< 0.001
**Induction of labour**	249 (25.5)	124 (25.1)	125 (25.9)	0.77

^a^sample size varies due to missing data; valid percentages are shown

^b^*IQR interquartile range*

There was no statistically significant difference in the prevalence of prenatal referral to an obstetrician between non-Western women and Dutch women (non Western women *n* = 247; 51% vs Dutch women, *n* = 227; 46%, *p* = 0.11). However, some reasons for prenatal referral did differ by ethnicity, which were gestational diabetes (Non-Western women; 17.2% versus Dutch women; 9.9%, *p* < 0.001) and malpresentation including breech position (Non-Western; 2.0% versus Dutch women; 8.8%, *p* < 0.001). In the not-prenatally referred group, 25 (5.0% of the not-prenatally referred women) cesarean sections were performed compared to 110 (23.2% of the prenatally referred women) in the prenatally referred group.

Finally, without adjusting for confounders, non-Western women did not differ in maternal and perinatal outcomes except for having, on average, a lower birthweight baby (small for gestational age (SGA): 12.2% of non-Western women versus 8.2% of Dutch women; large for gestational age (LGA): 5.2% versus 9.5%; *p* = 0.009) (Table 2).

**Table 2:** Maternal and perinatal outcomes of Dutch and non-Western women (*N* = 977)

	**Study population *N*= 977 (%)**^a^	**Dutch women *n* = 494 (50.6%)**^a^	**Non-Western non-Dutch women *n* = 483 (49.4%)**^a^	***P *value**
**Gestational age at the onset of labour**				0.71
Full-term-birth	920 (94.3)	467 (94.5)	453 (94.0)	
Preterm birth (< 37 weeks)	56 (5.7)	27 (5.5)	29 (6.0)	
**Mode of birth**				0.24
Spontaneous	747 (76.7)	367 (74.4)	380 (79.0)	
Vaginal assisted birth	92 (9.4)	52 (10.6)	40 (8.3)	
Cesarean section	135 (13.9)	74 (15.0)	61 (12.7)	
**Postpartum hemorrhage**	64 (6.7)	35 (7.2)	29 (6.1)	0.46
**Perineal status**				0.14
Intact	438 (51.8)	228 (53.8)	210 (49.9)	
Ruptured	243 (28.8)	109 (25.7)	134 (31.8)	
Episiotomy	164 (19.4)	87 (20.5)	77 (18.3)	
**Birth weight**^b^				0.009
SGA (< 10th percentile)	98 (10.2)	40 (8.2)	58 (12.2)	
AGA (p10-p90)	794 (82.4)	400 (82.3)	394 (82.6)	
LGA (> 90th percentile)	71 (7.4)	46 (9.5)	25 (5.2)	
**Perinatal death**	13 (1.3)	6 (1.2)	7 (1.4)	0.75
**Apgar score after 5 min**				0.36
< 7	22 (2.3)	9 (1.8)	13 (2.7)	

^a^sample size varies due to missing data; valid percentages are shown

^b^*SGA* small for gestational age, *AGA* average for gestational age, *LGA* large for gestational age

### Missing data

All variables had less than 5% missing, except for perineum, which had 13.5% missing observations.

Obstetricians who take over the care during the birth due to complications, do not always share all the information with the midwives directly after birth, resulting in missing observations. Missingness was not associated with ethnicity, thus did not bias the study's findings (Supplementary file 1).

### Regression analyses of the association between ethnicity and maternal and perinatal outcomes

Tables 3 and 4 demonstrate the crude and adjusted results of the logistic regression analyses. Potential effect modifiers, such as parity and prenatal referral, were assessed. The interaction of prenatal referral and ethnicity was only significant for the mode of birth, specifically the category caesarean section (OR 0.34, 95%CI 0.13–0.86). Therefore, mode of birth was stratified by prenatal referral (yes/no). No other interactions were statistically significant (interaction terms not shown).

**Table 3:** Unadjusted and adjusted associations of maternal and perinatal outcomes in women with a non-Western ethnicity compared to Dutch ethnicity

	**Unadjusted OR (95%CI)**	**Adjusted OR (95%CI)**
**Gestational age**^**a**^		
Term birth (≥ 37 weeks)	1.00 (ref)	1.00 (ref)
Preterm birth (< 37 weeks)	1.11 (0.65–1.90)	1.41 (0.76–2.63)
**Postpartum hemorrhage**^**b**^		
No	1.00 (ref)	1.00 (ref)
Yes	0.83 (0.50–1.38)	0.72 (0.42–1.25)
**Perineal status**^**c**^		
Intact	1.00 (ref)	1.00 (ref)
Lacerated	1.34 (0.97–1.83)	**1.59 (1.14–2.21)**
Episiotomy	0.96 (0.67–1.38)	1.29 (0.86–1.93)
**Birth weight**^**d,e**^		
AGA (p10-p90)	1.00 (ref)	1.00 (ref)
SGA (< p10)	1.47 (0.96–2.25)	1.46 (0.95–2.24)
LGA (> p90)	0.55 (0.33–0.92)	**0.50 (0.29–0.84)**

^a^Adjusted for: birth weight, parity

^b^Adjusted for: deprived area

^c^Adjusted for: parity, maternal educational level

^d^Adjusted for: BMI

^e^ *AGA* average for gestational age, *SGA* small for gestational age,* LGA* large for gestational age

No regression analyses for perinatal death and Apgar score due to low prevalence (< 5%)

Numbers in bold are statistically significant (*p* < 0.05)

**Table 4:** Mode of birth associated with non-Western ethnicity compared to Dutch ethnicity stratified by prenatal referral

	**Non-referred**		**Referred**	
	**Unadjusted OR (95%CI)**	**Adjusted OR (95%CI)**	**Unadjusted OR (95%CI)**	**Adjusted OR (95%CI)**
**Mode of birth**^**a**^				
Spontaneous	1.00 (ref)	1.00 (ref)	1.00 (ref)	1.00 (ref)
Vaginal assisted birth	0.61 (0.33–1.13)	0.76 (0.40–1.45)	0.88 (0.47–1.65)	1.02 (0.53–1.98)
Cesarean section	1.66 (0.73–3.78)	1.86 (0.81–4.26)	**0.58 (0.37–0.89)**	**0.63 (0.40–0.98)**

^a^Adjusted for: parity, age

Numbers in bold are statistically significant (*p* < 0.05)

Only the adjusted results that showed a significant association or a large effect size with ethnicity are discussed. Compared to Dutch women, non-Western women had 0.50 (95%CI 0.29–0.84) decreased odds of having a LGA baby while 1.46 (0.95–2.24) times the odds of having a SGA baby and 1.41 (0.76–2.63) times the odds of preterm birth, when adjusted for confounders (Table 3). Furthermore, when adjusted for confounders, non-Western women had 1.59 (1.14–2.21) increased odds of a perineal laceration compared to Dutch women.

Among women who were prenatally referred, non-Western women were less likely to have a caesarean section (aOR 0.63, 0.40–0.98) (Table 4). Among women who were not-prenatally referred, non-Western women were not significantly more likely to have a caesarean section than Dutch women (aOR 1.86, 0.81–4.26).

## Discussion

This study investigated how ethnicity is associated with maternal and perinatal outcomes in low-risk women in a midwife-led care setting and how maternal factors influence these relationships. Our findings propose that ethnicity is associated with some maternal and perinatal outcomes and that maternal factors mitigate or strengthen these associations. Our main finding is that non-Western women had increased odds of perineal laceration and decreased odds of high birthweight. In the prenatally referred group, non-Western women had decreased odds of caesarean section. After adjusting for the following confounders birthweight, parity, maternal educational level, deprived area and BMI, all odds ratios increased, except for the odds ratio of the association between ethnicity and birthweight, meaning that the maternal factors e.g., maternal educational level and parity, did not explain the differences between non-Western and Dutch women.

Compared to Dutch women, non-Western women were 1.5 times more likely to sustain perineal laceration, when adjusted for confounders. According to previous studies, Asian women have the highest risk of having severe perineal laceration compared to Western women, whereas Black women are less likely to have a perineal laceration [30–32]. The reasons behind these ethnic disparities are not well understood. Possible reasons may be differences in the perineum anatomy or tissue strength, or a prolonged second stage of labor [33, 34]. The results of our study are not in line with previous studies since, in our study, non-Westerns were more likely to sustain perineal laceration during labor [30–32]. Possible reasons for this difference could be that previous studies did not distinguish between low- and high-risk pregnant women. However, it is essential to note that 13.5% of the variable perineum was missing, which could have positively impacted the results of the association between perineum and ethnicity.

According to our study, non-Western women were significantly less likely to give birth to a baby with a high birthweight; this is in line with prior research conducted in both high- and low-risk women [35, 36]. Ethnic disparities in birthweight at term may be mainly due to normal biological variation, such as differences in maternal and paternal height [37–40]. In a study conducted by Troe et al. on ethnic differences in birthweight, non-Western women were not only significantly more likely to be shorter in height, also maternal and paternal weight partially explained the ethnic differences found in birthweight [41]. Biological variation could partially explain why non-Western women were less likely to give birth to a child with high birthweight than Dutch women despite the increased incidence of gestational diabetes and excessive weight gain among the non-Western group. Our findings support customised growth charts that include information on, among others, ethnicity, maternal and paternal height and maternal weight for monitoring the growth of the fetus [42].

Prenatally referred non-Western women were significantly less likely to undergo caesarean section than prenatally referred Dutch women. In the non-referred group, a caesarean section could still be performed if the pregnant woman was referred during labor. In this non-referred group, there were 5.0% caesarean sections. Non-Western women were twice as likely to undergo a caesarean section, but we did not find a significant association. This non-significant result may be due to the low percentage of caesarean sections in the non-referred group (5%), which is in line with previous studies in which intervention during birth were seen less in low-risk women compared to high risk women [13]. A study conducted by Edmonds et al. in low-risk pregnant women showed that non-Western women are more likely to undergo caesarean section due to for example fetal distress [43]. These results are in in line with other studies conducted in both low- and high-risk pregnant women [1, 44, 45]. This might explain the relatively higher caesarean section rate in non-referred non-Western women. The fact that in our study referred Dutch women were more likely to have a caesarean section than referred non-Western women, even though non-Western were more often referred, might be due to factors other than the medical characteristics of the client. Prior research has shown that midwife's referral behaviour and non-medical characteristics, such as ethnicity and deprivation, also play a role [46, 47]. More research is needed to elucidate the possible ethnic disparity in referral.

Contrary to other research, maternal factors did not explain the association between ethnicity and maternal and perinatal outcomes [4, 48]. A possible reason why maternal factors did not explain ethnic differences could be that there are still relatively unknown or unexplored maternal factors, for example, epigenetics, that could not be researched in this study [40, 49]. Another reason could be that maternal factors, such as parity, were protective factors in this study population. For example, multiparity has been shown to be associated with a lower chance of perineal laceration [50]. In this study, non-Western women were more likely to be multiparous. The fact that multiparity is more common among non-Western women in this study could explain why the odds ratios increased after adjusting for parity.

Overall, similar to research done in high-risk pregnant women, low-risk non-Western pregnant were in this study also disproportionally burdened by adverse maternal and perinatal outcomes.

## Strengths, limitations, and recommendations

This study is the first to analyse the associations between ethnicity and various maternal and perinatal outcomes among low-risk women in midwife-led care. This study used a near-complete dataset. There were almost no missing values, except for the variable perineum. This dataset included a broad spectrum of medical, socioeconomic, lifestyle, and psychosocial information, making adjusting for possible confounders feasible. Furthermore, midwives collected data, leading to a uniform policy concerning data collection and high reliability.

However, our research has its limitations. The operationalization of ethnicity only consisted of two categories: Dutch and non-Western. However, a growing body of literature indicates that variations within the non-Western community also exist [3, 31, 32, 34, 51–53].

Another limitation is that the dataset did not operationalize socioeconomic status. In our study, the variables deprived area and maternal educational level were used as a proxy for socioeconomic status, as multiple studies showed that deprived area and education level are associated with maternal and perinatal outcomes [54–56]. In the literature, more variables are used as a proxy for socioeconomic status; however, we believe that by including deprived areas and maternal educational levels in our analyses, we have managed to get sufficient insight into the socioeconomic status of the participants.

In the Dutch obstetric care system, health workers can make use of interpreters, with all costs fully reimbursed, in case of a language barrier [10]. Furthermore information about maternal and newborn care can be provided in various languages. However, our results suggest that there is still a need to create new strategies for the prevention of adverse perinatal outcomes among non-Western women. The focus should be on educating women on the obstetric care system, making care more culturally appropriate for ethnic minorities and improving the socioeconomic and sociodemographic position of the pregnant women. Prior research has shown that cultural factors, language barriers or overall lack of knowledge of the maternity care system and sociodemographic and socioeconomic factors can discourage and impede the use of maternity care services by non-Western women [12]. A Dutch study program called Healthy Pregnancy 4 All also showed that in order to improve maternal and neonatal health care, extensive collaboration is needed between the government and health sectors as health disparities are both medical and social [57].

More research, using national data, is needed to confirm our findings and estimate the association between perinatal death, low Apgar score and ethnicity. Furthermore, future studies should investigate the various ethnic groups within the non-Western community. Moreover, more research is needed to elucidate the possible ethnic disparity in referral behavior of Midwifery centers.

## Conclusions

Maternal and perinatal outcomes differed between low-risk non-Western and Dutch women in a midwife-led care setting. A perineal laceration occurred more often among non-Western women, and fewer children with high birthweights were born. Non-Western ethnicity was negatively associated with caesarean section in the prenatally referred group.

## Supplementary Information

Supplementary Material 1.

## Data Availability

The datasets used and analysed for this study are available from the corresponding author on reasonable request.

## Abbreviations

KI-R: The Kotelchuck Index-Revised
CIE: Change-in-estimate criterion
SGA: Small for gestational age
LGA: Large for gestational age
AGA: Appropriate for gestational age
