# Supplementary material for: Differences in maternal and perinatal outcomes between Dutch and non-Western women in a midwife-led care setting: a retrospective cohort study
Source: BMC Pregnancy Childbirth. 2024 Nov 29;24:803. doi: 10.1186/s12884-024-06982-2 (PMC11605869; doi:10.1186/s12884-024-06982-2)
Supplement: Supplementary file 1 — Supplementary Material 1. [file 12884_2024_6982_MOESM1_ESM.docx]

**Supplementary File**

**Table S1. Prevalence of missing data.**

| **Type** | **Covariate** | **n** | **%** |
| --- | --- | --- | --- |
| **Outcome** | Gestational age at onset of labour | 1 | 0.1 |
|  | Mode of birth | 3 | 0.3 |
|  | Postpartum hemorrhage | 16 | 1.6 |
|  | Perineal status | 132 | 13.5 |
|  | Birthweight | 14 | 1.4 |
|  | Perinatal death | 0 | 0 |
|  | Apgar score after 5 min | 5 | 0.5 |
| **Exposure** | Ethnicity | 0 | 0 |
| **Maternal characteristics** | Age | 0 | 0 |
|  | Prenatal care utilization | 0 | 0 |
|  | Prenatal referral to the obstetrician | 0 | 0 |
|  | Level of education | 26 | 2.7 |
|  | Deprived area | 0 | 0 |
|  | Parity | 0 | 0 |
|  | Psychosocial problems | 2 | 0.2 |
|  | Smoking | 3 | 0.3 |
|  | Alcohol use | 3 | 0.3 |
|  | Body mass index | 2 | 0.2 |
|  | Pregnancy-induced hypertension | 0 | 0 |
|  | Gestational diabetes | 0 | 0 |
|  | Induction of labour | 1 | 0.1 |
